# A 3.0-V, High-Precision, High-PSRR BGR with High-Order Compensation and Improved FVF Pre-Regulation

**DOI:** 10.3390/mi16121405

**Published:** 2025-12-14

**Authors:** Yongkang Shen, Jianhai Yu, Fading Xiao, Chang Cai, Chao Wang, Jinghu Li, Caiyan Ma, Yonghao Mo

**Affiliations:** 1 School of Computer, Electronics and Information, Guangxi University, Nanning 530004, China; 2313393046@st.gxu.edu.cn (Y.S.); 2413392001@st.gxu.edu.cn (C.C.); 2413391025@st.gxu.edu.cn (C.W.); 2 The Guangxi Key Laboratory of Machine Vision and Intelligent Control, Wuzhou University, Wuzhou 543002, China; 18177749417@163.com (C.M.); 15240708514@163.com (Y.M.); 3 School of Information and Communication, Guilin University of Electronic Technology, Guilin 541004, China; 13729054792@163.com; 4 The College of Computer and Information Sciences, Fujian Agricultural and Forestry University, Fuzhou 350002, China; lily_hit@126.com

**Keywords:** bandgap reference, temperature coefficient, power supply rejection ratio, base current correction, high-order compensation, flipped voltage follower

## Abstract

A 3.0 V bandgap reference (BGR) for battery management integrated circuit (BMIC) is presented, achieving a low temperature coefficient (TC) and a high power supply rejection ratio (PSRR). Precision is enhanced through two techniques: (1) a base current correction technique eliminates errors from the bipolar junction transistor (BJT) base current, and (2) a high-order temperature compensation circuit counteracts the inherent nonlinearity of the BJT’s base-emitter voltage (*V_BE_*). Furthermore, an improved flipped voltage follower (FVF) pre-regulation structure is integrated for efficient power supply noise suppression. The circuit is designed based on a 180 nm BiCMOS process, occupying a layout area of 0.0459 mm^2^. Post-layout simulation results demonstrate that the BGR achieves a temperature coefficient of 1.59 ppm/°C over the −40 °C to 125 °C temperature range. Within a supply voltage range of 4.7 V to 5.3 V, the line regulation is 0.00058 mV/V. At a 5.0 V supply voltage, the quiescent current is 23 μA, and the PSRR is −128.89 dB@1 Hz and −102.9 dB@1 kHz.

## 1. Introduction

The signal processing accuracy of high-performance analog systems, such as sensor circuits and BMICs, is highly dependent on a stable and precise voltage reference [1,2]. In automotive electronics applications, the core task of a BMIC is to continuously monitor the voltage and current of lithium-ion (Li-ion) battery arrays, which typically operate from 3.0 V to 4.0 V, and to precisely indicate their state of charge (SoC) [3]. The analog-to-digital converter (ADC), which converts the analog battery voltage to a digital format, fundamentally relies on the precision of its voltage reference for conversion accuracy [4]. To consistently meet the stringent system-level measurement accuracy (e.g., ±3 mV) across the wide operating temperature range of −40 °C to 125 °C, the voltage reference’s temperature coefficient needs to be suppressed to below 5 ppm/°C [5].

The bandgap reference (BGR) is the most prevalent topology for generating a reference source insensitive to temperature and supply voltage variations. However, due to the inherent high-order nonlinearity in the BJT’s base-emitter voltage, circuits employing only first-order temperature compensation are limited to a best-case TC approaching 10 ppm/°C [6,7,8,9,10], which is insufficient for high-precision applications. To achieve a lower TC, higher-order temperature compensation techniques have become the requisite approach. Consequently, numerous circuit topologies for high-order compensation have been reported [11,12,13,14,15,16]. The circuit in [11] compensates for the nonlinear term using a current with an analytical form of *T*ln(*T*), achieving a voltage reference as low as 0.706 ppm/°C. However, this comes at the cost of a complex topology and a high quiescent current consumption of 409 μA. A piecewise curvature compensation technique is proposed in [12]. This method utilizes comparators and digital logic gates to partition the entire temperature range into 11 regions, injecting a different compensation current for each. While it achieves a simultaneous excellent TC of 1.08 ppm/°C and an extremely low power consumption of 918 nW, this comes at the cost of an exceedingly complex circuit structure.

The output voltage of conventional bandgap references is approximately 1.2 V. To satisfy the demand for high-voltage references in applications such as battery management, numerous schemes combining an output voltage greater than 2 V with high-order temperature compensation have been recently proposed [3,4,5,17,18,19]. A cross-connected NPN topology is proposed in [4] to achieve a higher output voltage, and the temperature coefficient is reduced to 2.23 ppm/°C via a piecewise exponential curvature correction technique. However, its PSRR performance is relatively moderate. A current-mode BGR is presented in [18] that implements high-order correction by combining multiple compensation techniques (including logarithmic, leakage, and piecewise curvature compensation). Its merits include an extremely low TC of 0.42 ppm/°C and a 2.5 V high output. However, the limitation of this design lies in its exceeding complexity; it not only relies on chopping and notch filters to eliminate offset induced by the operational transconductance amplifier (OTA) but also employs a *β*-compensation technique to counteract the output’s proportional-to-absolute-temperature (PTAT) and non-PTAT fluctuations. Compared with the piecewise exponential curvature correction technique in [4] and the multi-technique architecture in [18], this work utilizes a streamlined topology to achieve a superior complexity-performance trade-off.

The automotive electronics environment is permeated with strong power supply noise originating from engines, inverters, and switching power supplies. Consequently, the voltage reference must also exhibit an extremely high PSRR to prevent this noise from coupling into high-precision measurement results. Researchers have explored various circuit techniques to address this power supply noise coupling and achieve high PSRR. The works in [20,21,22] utilize pre-regulation techniques to actively filter noise from the external supply, providing a clean, isolated internal supply for the core BGR circuit, thereby significantly enhancing the suppression of power supply noise. The work in [23] employs the collector common-mode voltage extraction feedback (CVEF) architecture and the all-sub-threshold-region low line sensitivity (ASLS) circuit to achieve high PSRR and low line sensitivity. A nonlinear current compensation technique is proposed in [24], which significantly mitigates supply variations and suppresses high-frequency power supply ripple. In [25], PSRR is improved by a coupling structure of the power supply and a feedback loop of the start-up circuit.

To satisfy the stringent requirements for temperature stability and power supply noise suppression in high-precision applications like BMICs, this paper presents a low-TC, high-PSRR voltage reference with a 3.0 V output. The circuit is implemented in a 180 nm BiCMOS process, utilizing its 5 V devices to be compatible with a 5.0 V supply voltage. The design employs a base current correction technique, effectively eliminating the errors introduced by the BJT base current. On this basis, by integrating a high-order temperature compensation circuit, the reference voltage’s TC is further optimized to 1.59 ppm/°C over the −40 °C to 125 °C temperature range. Addressing the critical PSRR performance, this paper also proposes and integrates an improved FVF structure. This FVF circuit, acting as an efficient pre-regulator, provides a highly isolated internal supply for the bandgap core, thereby ensuring the circuit’s excellent power noise suppression capability. The remainder of this paper is organized as follows. Section 2 introduces the proposed voltage reference circuit structure and its principles. Section 3 presents the post-layout simulation results of the proposed circuit. Finally, Section 4 concludes the paper.

## 2. Proposed Circuit Structure and Principles

The conventional first-order bandgap reference circuit generates a temperature-insensitive reference output by combining voltages with complementary temperature characteristics from BJTs. Specifically, the base-emitter voltage (*V_BE_*) of a BJT exhibits a negative temperature coefficient, making it complementary to absolute temperature (CTAT). In contrast, the difference in *V_BE_* between two BJTs operating at different current densities (Δ*V_BE_*) produces a voltage that is proportional to absolute temperature (PTAT). Although the negative temperature characteristic of *V_BE_* is predominantly linear, its inherent higher-order physical effects introduce a nonlinear temperature curvature, which is accurately described by its complete physical model [26]:

(1) $$V_{BE}(T)=V_{G0}(T_{r})-\left[V_{G0}(T_{r})-V_{BE0}(T_{r})\right]\frac{T}{T_{r}}-(\eta -\theta )V_{T}\ln(\frac{T}{T_{r}}),$$

where *V_G_*_0_(*T_r_*) is the extrapolated bandgap voltage at the reference temperature *T_r_*, *η* is a temperature- and process-independent constant (typically 3.54), and *θ* represents the temperature-dependent order of the collector current. *V_T_* = *kT*/*q* is the thermal voltage, where *k* is the Boltzmann constant, *T* is the absolute temperature, and *q* is the electron charge. The final term denotes the nonlinear temperature dependency of *V_BE_*.

**Figure 1:**
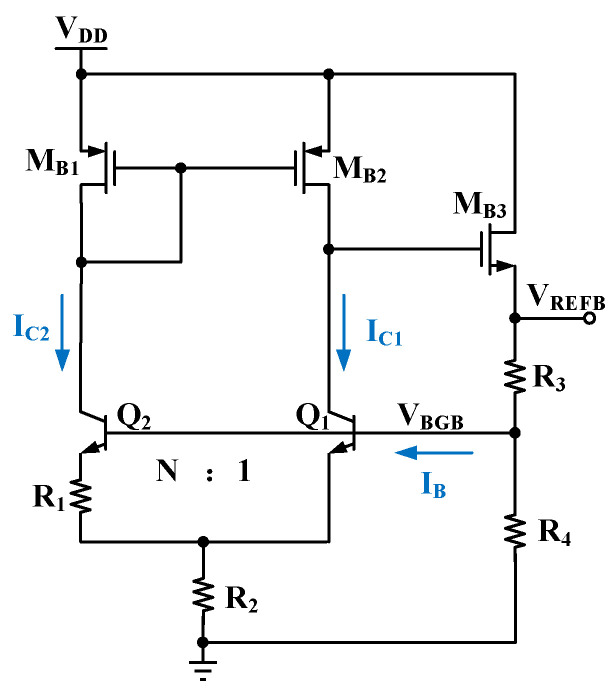
Schematic of the Brokaw reference with a higher output voltage.

First-order temperature compensation is achieved by summing the *V_BE_*, which has a negative temperature coefficient, with an appropriately weighted voltage Δ*V_BE_* that exhibits a positive temperature coefficient. The resulting bandgap reference voltage is typically stabilized at approximately 1.2 V. However, many applications, such as battery management chips, require a higher voltage. The schematic of the Brokaw BGR [27], which outputs a higher voltage, is shown in Figure 1. By connecting the bandgap voltage (*V_BGB_*) to a resistor division network composed of R_3_ and R_4_, a reference voltage (*V_REFB_*) higher than *V_BGB_* can be generated. The formula for the bandgap voltage *V_BGB_* in this structure is:

(2) $$V_{BGB}=V_{BE1}+2\frac{R_{2}}{R_{1}}V_{T}\ln(N\frac{I_{C1}}{I_{C2}})\approx V_{BE1}+2\frac{R_{2}}{R_{1}}V_{T}\ln(N),$$

where *N* is the emitter area ratio of Q_2_ to Q_1_, *I_C_*_1_ is the collector current of Q_1_, and *I_C_*_2_ is the collector current of Q_2_. Assuming zero mismatch in the current mirror, *I_C_*_1_ and *I_C_*_2_ can be considered approximately equal. The base currents of Q_1_ and Q_2_ flow through the resistor R_3_, thereby introducing an additional temperature-dependent factor into the reference voltage *V_REFB_*. The formula for the reference voltage *V_REFB_* is:

(3) $$V_{REFB}=(1+\frac{R_{3}}{R_{4}})V_{BGB}+I_{B}R_{3}$$

Assuming *V_BGB_* is temperature-independent, the TC of *V_REFB_* becomes directly dependent on the base current *I_B_*, which exhibits an exponential relationship with temperature. This *I_B_R*_3_ term introduces an additional and undesirable temperature drift error into *V_REFB_*, which complicates the temperature compensation of the final output voltage.

### 2.1. Design Considerations

**Figure 2:**
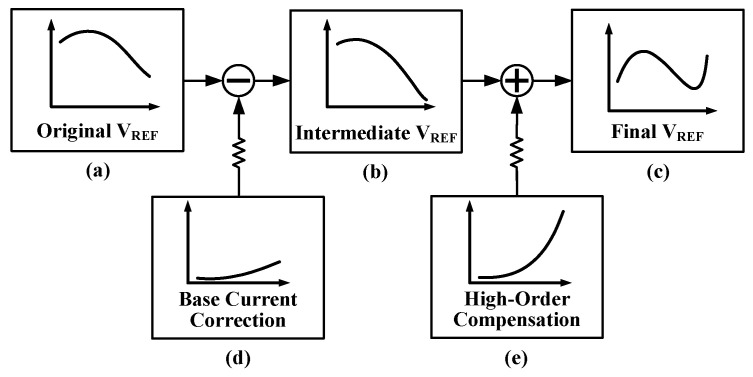
(**a**) First-order reference voltage affected by base current; (**b**) First-order reference voltage after base current correction; (**c**) Reference voltage after high-order compensation; (**d**) Base current correction; (**e**) Curve of high-order compensation current.

This paper proposes a base current correction technique, which eliminates the influence of base current on the reference voltage by introducing a correction current and a correction resistor. The trend of the first-order compensated voltage *V_REF_* is shown in Figure 2a, which is affected by both the base current and the resistor division network. Through the proposed base current correction, the temperature sensitivity of this reference voltage is optimized from the curve shown in Figure 2a to the ‘pure’ first-order reference voltage shown in Figure 2b, which is unaffected by base current.

The strategy for achieving high-order compensation in this work is to ensure the temperature variation of the *V_REF_* curve (after base current correction) is minimized in the lower part of the temperature range, as shown in Figure 2b. Then, an upward-curving high-order compensation current (curve shown in Figure 2e) is introduced to achieve the final low-TC *V_REF_*, as depicted in Figure 2c.

Furthermore, this paper proposes an improved FVF structure, designed to enhance the PSRR of the reference voltage. The voltage generated by this improved FVF structure is used as the supply for the bandgap core circuit, thereby significantly improving the PSRR of the output reference voltage.

**Figure 3:**
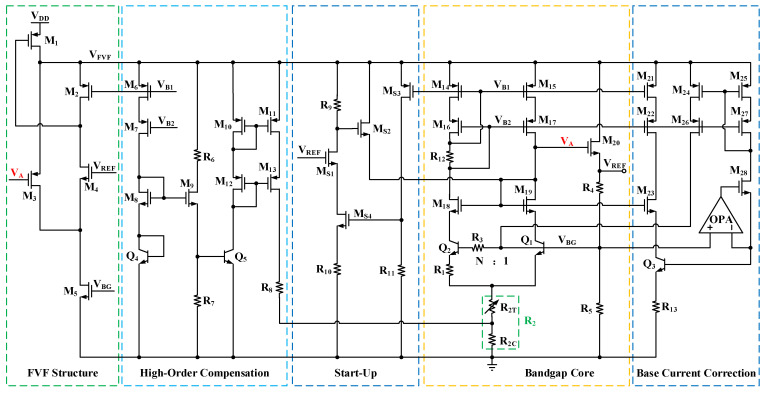
Schematic of the proposed high-output-voltage reference circuit.

Figure 3 illustrates the schematic of the proposed high-output-voltage reference circuit, which comprises an FVF structure, high-order compensation, a start-up circuit, a bandgap core, and base current correction.

### 2.2. Bandgap Reference with Base Current Correction

**Figure 4:**
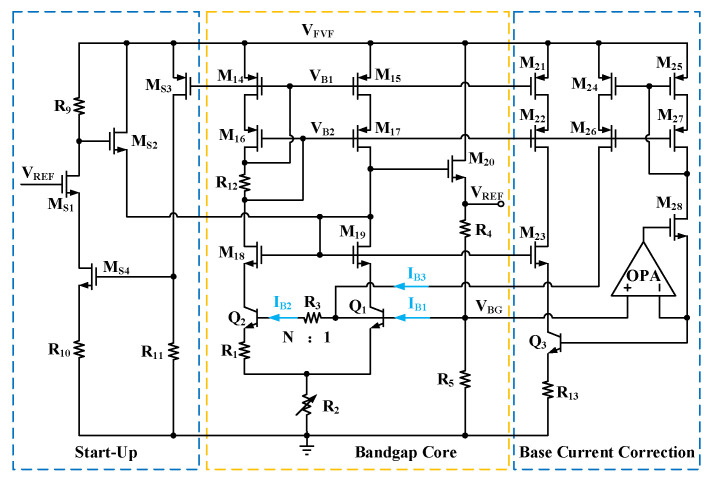
Schematic of bandgap core circuit with base current correction and start-up.

Figure 4 illustrates the schematic of the proposed bandgap core circuit, which incorporates a base current correction scheme and a start-up circuit. The design is based on the classic Brokaw bandgap topology, where the internally generated bandgap voltage, *V_BG_*, is connected to a resistive divider network formed by *R*_4_ and *R*_5_. To achieve precise current replication and biasing, the circuit integrates a set of PMOS low-voltage cascode current mirrors and a set of NMOS current mirrors. Specifically, the PMOS transistors M_14_–M_17_, along with the biasing resistor R_12_, constitute a low-voltage cascode structure with high output impedance, designed to enhance current matching accuracy and suppress the channel-length modulation effect. Correspondingly, the NMOS transistors M_18_ and M_19_ form a basic current mirror to perform the required current mirroring. The first-order compensated *V_BG_* is given by Equation (2). The base currents of Q_1_ (*I_B_*_1_) and Q_2_ (*I_B_*_2_), along with a correction current (*I_B_*_3_), flow through resistor *R*_4_, generating a voltage drop that introduces additional temperature-dependent terms. Consequently, the first-order reference voltage, *V_REF_*_1_, is expressed as:

(4) $$V_{REF1}=(1+\frac{R_{4}}{R_{5}})V_{BG}+(I_{B1}+I_{B2}-I_{B3})R_{4}$$

According to Equation (4), assuming that *V_BG_* is ideally a temperature-independent value, the temperature coefficient of *V_REF_*_1_ is then dependent on the total base current. Based on the physical model of BJTs, the base current can be expressed as *I_B_* = *I_C_*/*β*(*T*). In this expression, *β*(*T*) = *β*_∞_exp(−Δ*E_G_*/*kT*), where *β*_∞_ is the maximum current gain of the transistor, and Δ*E_G_* is the bandgap narrowing factor of the emitter, both of which are temperature-independent. Therefore, *I_B_* is an exponential function of temperature.

The offset voltage on *V_REF_*_1_ can be corrected by introducing a correction resistor, R_3_, between the bases of Q_1_ and Q_2_. The base current *I_B_*_2_ flows through *R*_3_, creating a voltage drop across it. This voltage drop adjusts the internal bandgap voltage *V_BG_*, which in turn further compensates *V_REF_*_1_. The resulting bandgap voltage, *V_BG_*, is given as:

(5) $$V_{BG}=V_{BE1}+\frac{2R_{2}(\Delta V_{BE}-I_{B2}R_{3})}{R_{1}}$$

By substituting Equation (5) into Equation (4), the reference voltage *V_REF_*_1_ can be expressed as:

(6) $$V_{REF1}=(1+\frac{R_{4}}{R_{5}})\left[V_{BE1}+2\Delta V_{BE}\frac{R_{2}}{R_{1}}-2I_{B2}R_{3}\frac{R_{2}}{R_{1}}+(I_{B1}+I_{B2}-I_{B3})\frac{R_{4}R_{5}}{R_{4}+R_{5}}\right]$$

For ease of the subsequent analysis, several temperature-independent ratio parameters are introduced: the current ratios are set as *K_A_* = *I_B_*_1_/*I_B_*_2_ and *K_B_* = *I_B_*_3_/*I_B_*_2_, while the resistor ratios are defined as *K_C_* = *R*_2_/*R*_1_ and *K_D_* = *R*_4_/*R*_5_. With these definitions, Equation (6) can be reformulated as:

(7) $$V_{REF1}=(1+K_{D})\left[V_{BE1}+2K_{C}\frac{kT\ln(N)}{q}+(R_{4}\frac{1+K_{A}-K_{B}}{1+K_{D}}-2K_{C}R_{3})\frac{I_{C2}}{\beta_{\infty }}\exp(\frac{\Delta E_{G}}{kT})\right]$$

As indicated by Equation (7), parameter *K_D_* dictates the level of the output reference voltage, whereas the first-order temperature coefficient is configured by *K_C_* and *N*. Due to differences in the emitter areas of Q_1_ and Q_2_ and their corresponding *V_BE_*, their current gain *β* also differs slightly, which leads to a mismatch in base currents. By accurately matching *I_B_*_3_ to *I_B_*_1_ such that *K_A_* = *K_B_*, the impact of the *β* and *V_BE_* variations between Q_1_ and Q_2_ can be effectively avoided. With parameters *K_A_* to *K_D_* established, the influence of the Q_1_ and Q_2_ base currents on the first-order reference *V_REF_*_1_ is then corrected by choosing a suitable *R*_3_ value that nullifies the $R_{4}\frac{1+K_{A}-K_{B}}{1+K_{D}}-2K_{C}R_{3}$ term.

**Figure 5:**
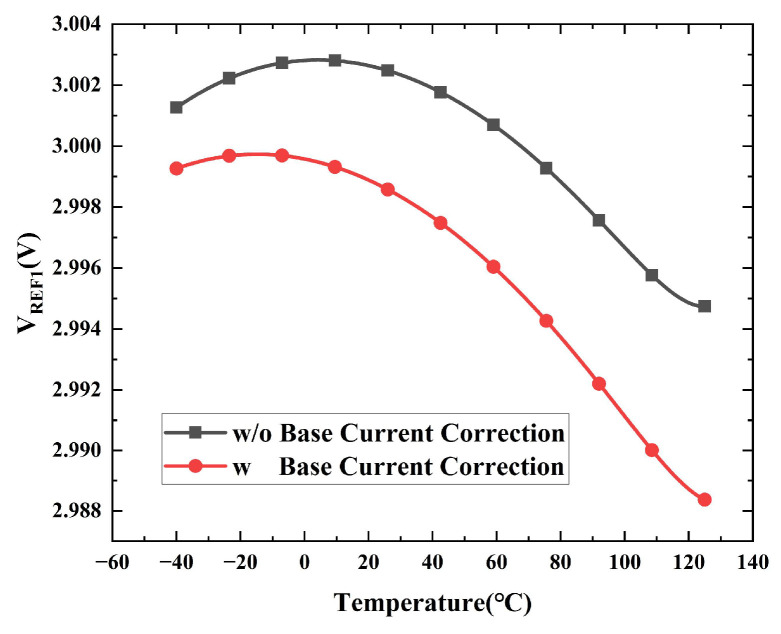
Simulated results of *V_REF_*_1_ without and with the base current correction.

The base current correction circuit proposed herein is devised to generate a correction current, *I_B_*_3_, that precisely matches *I_B_*_1_. It consists of a replica BJT Q_3_, an operational amplifier (OPA), MOSFETs M_21_–M_28_, and resistor R_13_. Transistors M_21_–M_23_ replicate the collector current of Q_1_ into Q_3_. Concurrently, the OPA forces the base of Q_3_ to the same potential as Q_1_’s base (*V_BG_*). With the resistance of R_13_ set to twice that of R_2_, the emitter voltage of Q_3_ is also made identical to that of Q_1_. As Q_3_’s collector current, base voltage, and emitter voltage are all forced to match those of Q_1_, it effectively becomes a perfect replica. Therefore, its base current serves as a high-fidelity copy of *I_B_*_1_. This current is then steered back to the BGR core via the low-voltage cascode current mirror (M_24_–M_27_) to perform the correction. Figure 5 shows a simulated comparison of *V_REF_*_1_ as a function of temperature, contrasting the cases without and with the base current correction. The results reveal that the uncorrected *V_REF_*_1_ exhibits an undesirable upward bow due to the base current effect. With the correction engaged, the reference voltage is restored to a much cleaner parabolic curve, which is the typical characteristic of a first-order bandgap reference.

The implemented start-up circuit, comprising transistors M_S1_–M_S4_ and resistors R_9_–R_11_, operates as follows. Initially, upon power-up, the absence of current in R_9_–R_11_ holds the gate-source voltage of M_S4_ low. This momentarily activates M_S2_, which injects a pulse of current into the BGR core to guarantee a reliable start-up. As the supply voltage ramps up, M_S3_ is turned on, raising the gate potential of M_S4_. Consequently, both M_S1_ and M_S4_ conduct and shut down M_S2_. Once M_S2_ is off, the start-up sequence is complete, and the BGR circuit settles into its normal steady-state operation.

### 2.3. High-Order Compensation

**Figure 6:**
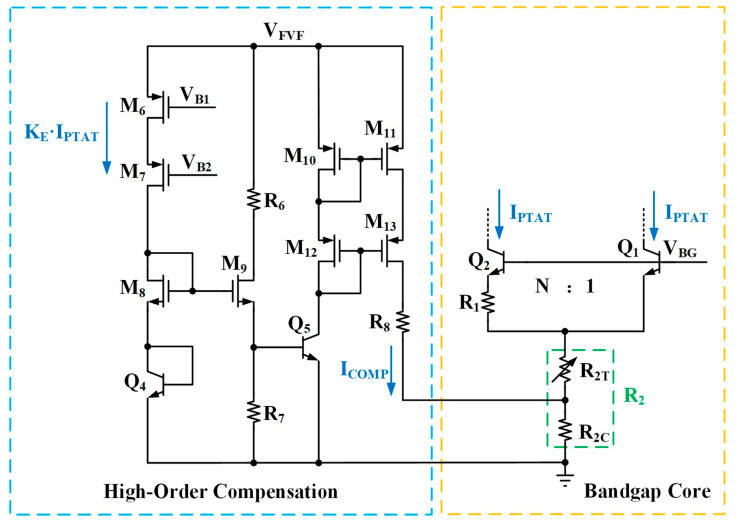
Schematic of the high-order compensation circuit.

While the previously described base current correction effectively removes the associated error, the ultimate TC performance remains governed by the inherent curvature of the *V_BE_*. Equation (1) reveals that *V_BE_* comprises a linear component and a high-order term *T*ln(*T*/*T_r_*). The latter is generally disregarded in first-order designs but becomes a critical consideration for achieving a low TC. To address this nonlinearity, this work introduces the high-order compensation circuit depicted in Figure 6. Here, transistors M_6_ and M_7_, part of two distinct current mirror pairs, are tasked with mirroring the core’s collector current (*I_PTAT_*) by a factor of *K_E_*. Consequently, the current through M_8_ is given by:

(8) $$I_{D8}=K_{E}\cdot \;I_{PTAT}=K_{E}\cdot \;\frac{V_{T}\ln(N)}{R_{1}}$$

The transistors M_8_ and M_9_ share the same width-to-length ratio, denoted as (*W_m_*/*L_m_*), and the BJTs Q_4_ and Q_5_ have identical emitter areas. Q_4_ is diode-connected, with its collector tied to its base. The resistors R_6_ and R_8_ serve to reduce the effect of channel-length modulation. According to the circuit, we can establish the relationship *V_GS_*_8_ + *V_BE_*_4_ = *V_GS_*_9_ + *V_BE_*_5_, where *V_GS_*_8_ and *V_GS_*_9_ are the gate-source voltages of transistors M_8_ and M_9_, respectively. From this, the following is derived:

(9) $$V_{GS8}-V_{GS9}=V_{BE5}-V_{BE4}$$

The MOSFETs of the proposed high-order compensation circuit operate in the saturation region. The current of M_8_ can be expressed as:

(10) $$I_{D8}=\frac{1}{2}\mu_{n}C_{ox}\frac{W_{m}}{L_{m}}{\left(V_{GS8}-V_{TH},n\right)}^{2}(1+\lambda_{n}V_{DS8}),$$

where *μ_n_* denotes the electron mobility, *C_ox_* is the gate oxide capacitance per unit area, *V_TH,n_* is the NMOS threshold voltage, *λ_n_* is the channel-length modulation parameter, and *V_DS_*_8_ is the drain-source voltage of M_8_. Assuming the source-to-body voltages (*V_SB_*) of M_8_ and M_9_ are nearly identical, their threshold voltages can be considered equal. Given that M_8_ and M_9_ have identical *W*/*L* ratios, and by ignoring the channel-length modulation effect, the NMOS saturation current equation yields:

(11) $$V_{GS8}-V_{GS9}=\sqrt{\frac{2L_{m}}{\mu_{n}C_{ox}W_{m}}}(\sqrt{I_{D8}}-\sqrt{I_{D9}})$$

The identical emitter areas of BJTs Q_4_ and Q_5_ yield the following:

(12) $$V_{BE5}-V_{BE4}=V_{T}\ln(\frac{I_{C5}}{I_{C4}})=V_{T}\ln(\frac{I_{D10}}{I_{D8}})$$

From Equations (9), (11), and (12), it can be derived that:

(13) $$I_{D10}=I_{D8}\cdot \;\exp(\frac{\sqrt{\frac{2L_{m}}{\mu_{n}C_{ox}W_{m}}}(\sqrt{I_{D8}}-\sqrt{I_{D9}})}{V_{T}})$$

The current flowing through transistor M_9_ can be expressed as:

(14) $$I_{D9}=I_{R7}+I_{B5}=\frac{V_{BE5}}{R_{7}}+\frac{I_{D10}}{\beta (T)}$$

In the circuit, transistors M_10_–M_13_ form a high-output-impedance cascode current mirror. This structure is employed to accurately replicate the collector current of Q_5_ and output it as the compensation current, *I_COMP_*, such that *I_COMP_* = *I_D_*_10_. This current is then fed into the bandgap core circuit to perform the required compensation function. By substituting Equations (8) and (14) into Equation (13), the expression for the compensation current *I_COMP_* is obtained as:

(15) $$I_{COMP}=\frac{K_{E}V_{T}\ln N}{R_{1}}\cdot \;\exp\left(\frac{\sqrt{\frac{2L_{m}}{\mu_{n}C_{ox}W_{m}}}}{V_{T}}(\sqrt{\frac{K_{E}V_{T}\ln N}{R_{1}}}-\sqrt{\frac{V_{BE5}}{R_{7}}+\frac{I_{COMP}}{\beta (T)}})\right)$$

**Figure 7:**
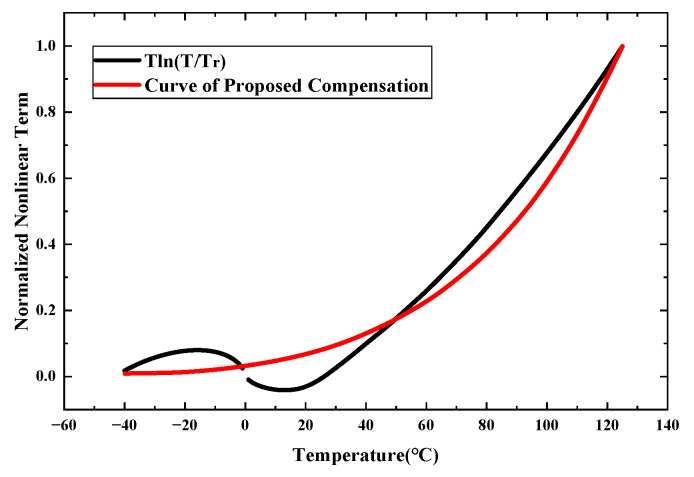
Normalized curves of *T*ln(*T*/*T_r_*) and Equation (15) using the estimated process parameters.

The values of the parameter *K_E_* and resistor *R*_7_ can be determined through numerical simulation using professional software such as MATLAB R2024b. For ease of analysis and visualization, the data were normalized. Figure 7 presents a comparison between the *T*ln(*T*/*T_r_*) term and the proposed compensation current curve. Within the temperature range from −40 °C to 125 °C, the generated compensation current effectively matches the *T*ln(*T*/*T_r_*) term.

The high-order compensation current *I_COMP_* is injected through the resistor R_2C_ into the bandgap core circuit, thereby achieving precise compensation for the nonlinear term. The reference voltage after high-order compensation can be expressed as follows:

(16) $$V_{REF}=(1+\frac{R_{4}}{R_{5}})(V_{BE1}+\frac{2R_{2}V_{T}\ln N}{R_{1}}+I_{COMP}\cdot \;R_{2C})$$

**Figure 8:**
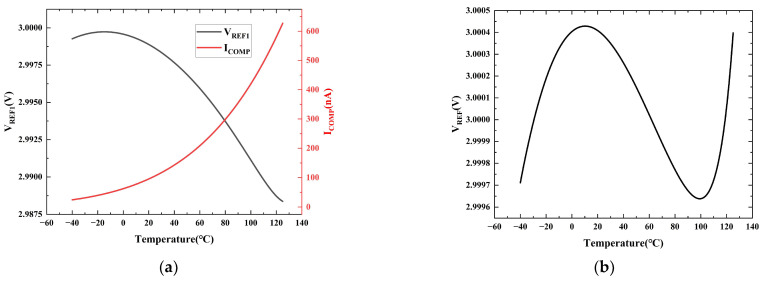
(**a**) Simulated *V_REF_*_1_ and *I_COMP_* versus temperature; (**b**) Simulated *V_REF_* versus temperature after high-order compensation.

Figure 8a plots the simulated temperature characteristics of the first-order reference voltage *V_REF_*_1_ and the compensation current *I_COMP_*. The simulation results show that the downward parabolic temperature drift of *V_REF_*_1_ exhibits a complementary relationship with the upward parabolic temperature drift of the precisely designed *I_COMP_*. Figure 8b presents the simulated temperature characteristics of the high-order compensated reference voltage *V_REF_*. After applying the high-order compensation, the temperature coefficient of the reference voltage is significantly reduced from 20.74 ppm/°C to 1.59 ppm/°C, which fully verifies the effectiveness of the proposed high-order compensation technique.

Furthermore, the stability of the proposed compensation network is inherently guaranteed by the system architecture. Unlike self-biased loops that may suffer from bias lock-up, the high-order compensation circuit is directly driven by the core’s *I_PTAT_* current via mirror transistors M_6_ and M_7_, eliminating the risk of zero-current states. Although the compensation current exhibits an exponential increase with temperature, simulation results verify that the high-order compensation circuit operates stably and continues to provide effective curvature correction at extended temperatures up to 130 °C, ensuring a safety margin beyond the specified 125 °C limit.

**Figure 9:**
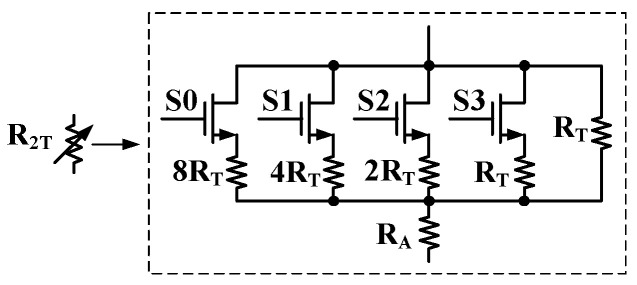
Schematic of the trimming circuit.

To enhance the robustness of the circuit design against process variations during fabrication, a trimming circuit is integrated into the proposed design. This circuit is intended to compensate for device parameter mismatches caused by process deviations, ensuring that the final circuit performance precisely converges to the design target. As shown in Figure 9, the trimming circuit consists of a 4-bit binary-weighted resistor array controlled by switches S3, S2, S1, and S0, where S3 represents the most significant bit, and S0 represents the least significant bit. A control code of “1” indicates that the switch is turned on, while “0” indicates that it is turned off. To minimize the impact of the switch on-resistance, the width-to-length ratio (*W*/*L*) of the NMOS transistors is designed to be very large (20 μm/500 nm). To achieve symmetric bidirectional trimming, the control code under the typical process corner is preset to the mid-range value “1000”, ensuring sufficient calibration margin for both fast and slow process corners. The resistance values of R_T_ and R_A_ are designed to be 24.5 kΩ and 200.5 kΩ, respectively. Post-layout simulation shows that a one-step change in the trimming code around the nominal setting results in a reference-voltage variation of approximately 2.7 mV. The full adjustment range provided by the 4-bit trimming network is sufficient to compensate for process-induced variations across all corners.

### 2.4. FVF Structure with Improved PSRR

**Figure 10:**
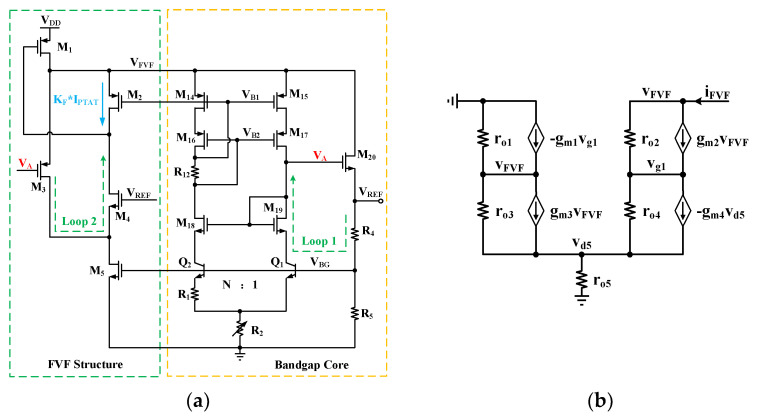
(**a**) Schematic of the FVF structure; (**b**) Equivalent output impedance model of the FVF structure.

A common method to improve the PSRR is to employ pre-regulation techniques, which provide a more stable intermediate supply voltage to isolate the core circuit from power supply noise. However, conventional pre-regulation approaches typically require additional biasing circuits. To address the *V_DD_* noise issue without significantly increasing power consumption and circuit complexity, a high-efficiency pre-regulation structure (an improved FVF structure) is proposed in this design, as shown in Figure 10a. The improved FVF structure is composed of transistors M_1_–M_5_, all of which operate in the saturation region to maintain sufficient gain and effective feedback regulation.

The key feature of this FVF topology lies in its self-referencing characteristic: it does not require an independent bias network, but instead reuses the stable voltages *V_A_*, *V_REF_*, and *V_BG_* generated by the bandgap core as its own reference, which are applied to the gates of M_3_, M_4_, and M_5_, respectively. By reusing these inherently stable node voltages, the improved FVF structure maintains its internal node voltages and operating currents largely independent of supply fluctuations. This creates an efficient cascaded regulation path that delivers a well-regulated internal supply, *V_FVF_*, to the bandgap core.

The proposed FVF structure does not feature a complex feedback network; its only negative feedback loop is formed through M_3_, M_4_, and M_1_ back to *V_FVF_*. Owing to its local negative feedback, the FVF structure achieves low output impedance [28,29], which helps suppress supply-induced noise in the following stages. Figure 10b shows the equivalent model of the output impedance for the proposed FVF structure, where *r_o_*_i_ and *g_m_*_i_ are the small-signal output resistance and transconductance of M_i_, respectively. Small-signal voltages and currents are denoted by lowercase letters. According to Kirchhoff’s laws, the following expressions can be derived:

(17) $$i_{FVF}+v_{d5}\frac{1}{r_{o3}}=v_{g1}(g_{m1}-\frac{1}{r_{o2}})+v_{FVF}(g_{m2}+g_{m3}+\frac{1}{r_{o1}}+\frac{1}{r_{o2}}+\frac{1}{r_{o3}}),$$

(18) $$v_{FVF}(g_{m2}+\frac{1}{r_{o2}})+v_{d5}(g_{m4}+\frac{1}{r_{o4}})=v_{g1}(\frac{1}{r_{o2}}+\frac{1}{r_{o4}}),$$

(19) $$v_{d5}(g_{m4}+\frac{1}{r_{o3}}+\frac{1}{r_{o4}}+\frac{1}{r_{o5}})=v_{FVF}(g_{m3}+\frac{1}{r_{o3}})+v_{g1}\frac{1}{r_{o_{4}}},$$

where *g_m_*_2_*r_o_*_2_ ≫ 1, *g_m_*_3_*r_o_*_3_ ≫ 1, *g_m_*_4_*r_o_*_4_ ≫ 1. Combining Equations (17)–(19), the output impedance of the FVF structure can be expressed as:

(20) $$r_{out}=\frac{v_{FVF}}{i_{FVF}}\approx \frac{1}{g_{m1}(g_{m2}+g_{m3})r_{o2}}$$

**Figure 11:**
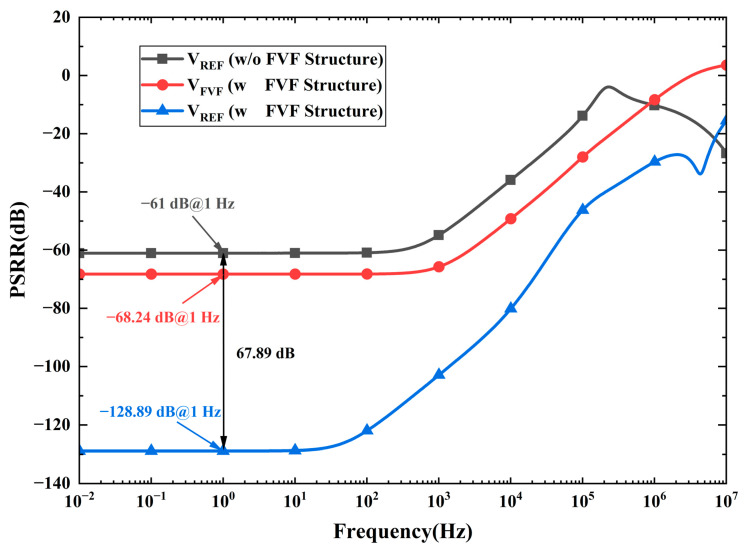
Comparison of PSRR performance without and with the FVF structure.

The output impedance of the proposed FVF structure is very low, effectively enhancing the PSRR of the bandgap core without extra power or biasing circuits. As shown in Figure 11, the simulation results indicate that the PSRR@1 Hz of *V_REF_* is improved by 67.89 dB after introducing the FVF structure compared to the case without it. This further verifies the effectiveness of the proposed FVF structure in enhancing PSRR.

## 3. Results

**Figure 12:**
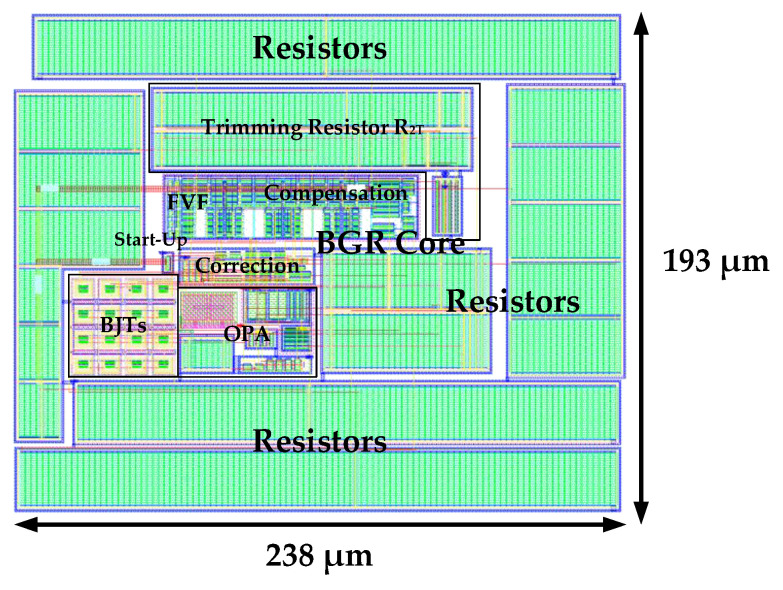
Layout of the proposed voltage reference.

Figure 12 shows the layout of the proposed voltage reference. Implemented in a standard 180-nm BiCMOS process, the circuit occupies an active area of 0.0459 mm^2^ (238 μm × 193 μm).

**Figure 13:**
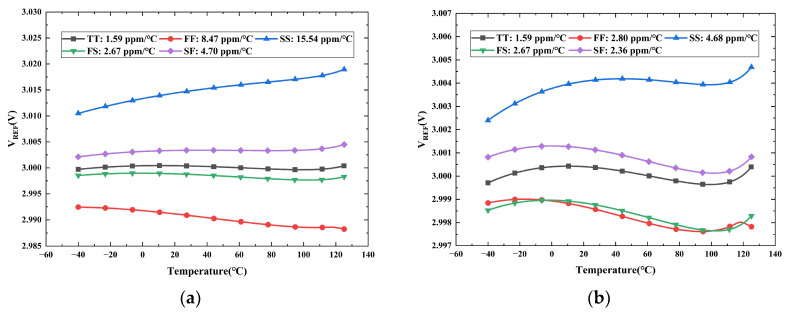
Simulated temperature dependence of *V_REF_* across five process corners: (**a**) Untrimmed; (**b**) Trimmed.

Figure 13a and Figure 13b show the post-layout simulation results of *V_REF_* versus temperature for the untrimmed and trimmed circuits, respectively. These simulations were conducted across five process corners (TT, FF, SS, FS, SF) under a 5 V supply from −40 °C to 125 °C, where T, F, and S represent typical, fast, and slow devices. As indicated by the results, the trimming process significantly minimizes the variation of *V_REF_* across the temperature range, yielding a much better TC performance compared to the untrimmed design.

**Figure 14:**
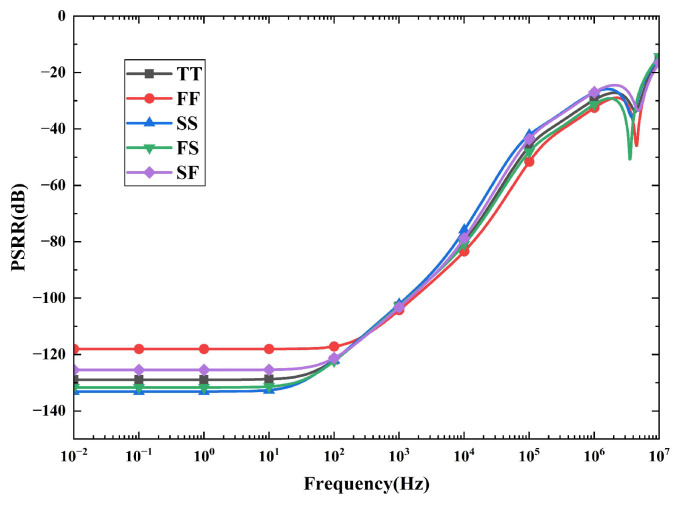
PSRR of *V_REF_* across different process corners.

Figure 14 illustrates the PSRR curves of *V_REF_* across different process corners at 27 °C with a supply voltage of 5 V. The circuit exhibits excellent power supply rejection performance at low frequencies. At 1 Hz, the PSRR is better than −118 dB across all process corners. Specifically, the PSRR values are −128.89 dB, −118.03 dB, −133.1 dB, −131.65 dB, and −125.45 dB for the TT, FF, SS, FS, and SF corners, respectively. As the frequency increases, the PSRR performance degrades. Taking the TT corner as an example, the PSRR remains at −102.9 dB at 1 kHz and decreases to −46.23 dB at 100 kHz.

**Figure 15:**
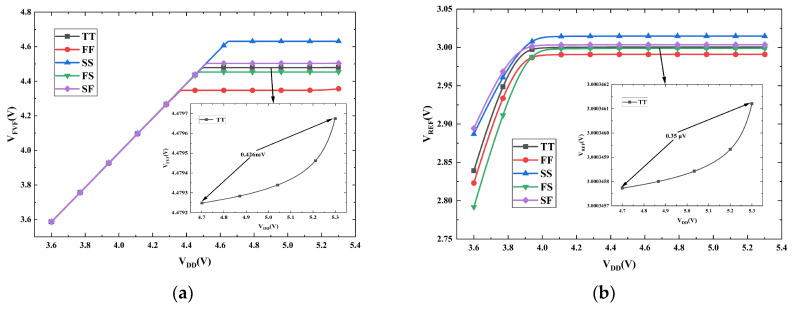
Simulated voltages versus supply voltage *V_DD_* across process corners: (**a**) *V_FVF_*; (**b**) *V_REF_*.

Figure 15a and Figure 15b illustrate the dependence of *V_FVF_* and *V_REF_* on the supply voltage (*V_DD_*), respectively. The simulations were conducted at 27 °C across five process corners. As observed in Figure 15a, *V_FVF_* enters a stable region when *V_DD_* exceeds 4.7 V. For a supply voltage range of 4.7 V to 5.3 V at the TT corner, the voltage variations of *V_FVF_* and *V_REF_* are 0.426 mV and 0.35 μV, respectively. Consequently, the calculated line regulation (LNR) for *V_FVF_* and *V_REF_* in this region is 0.71 mV/V and 0.58 μV/V, respectively.

**Figure 16:**
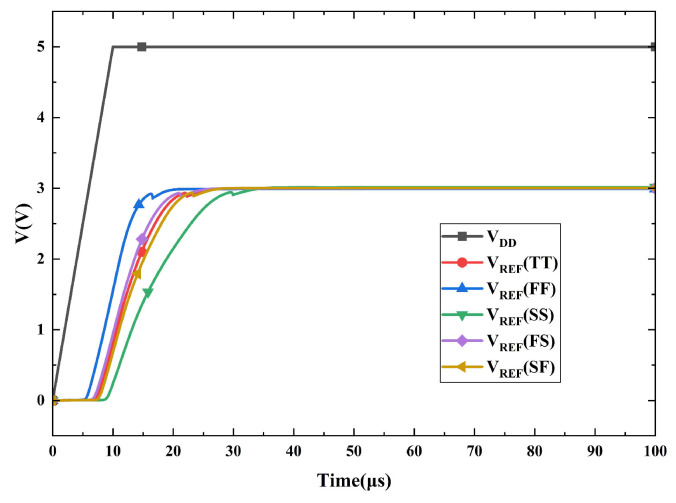
Simulated start-up response across five process corners.

The transient simulation results in Figure 16 demonstrate that the circuit functions correctly during the power-up sequence with a 10 μs supply ramp. *V_REF_* stabilizes at 3.0 V within 40 μs across all five process corners, validating the effectiveness of the proposed start-up circuit.

**Figure 17:**
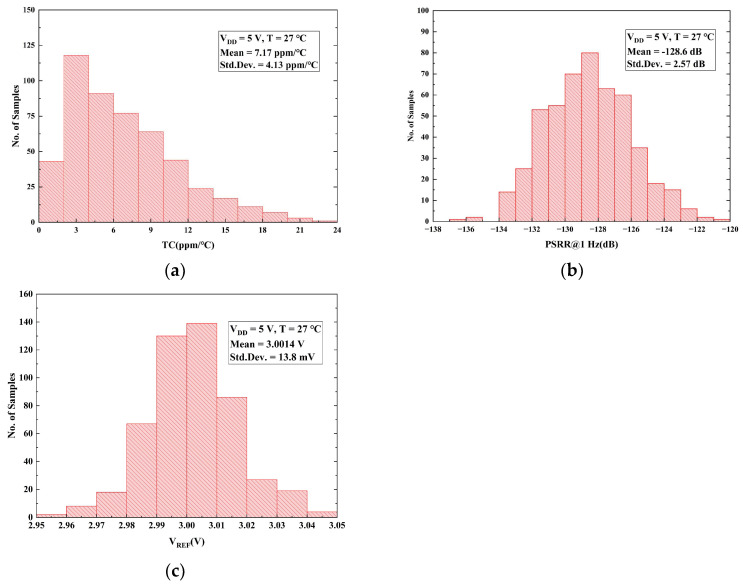
Monte Carlo simulation results over 500 runs: (**a**) TC; (**b**) PSRR@1 Hz; (**c**) *V_REF_*.

Figure 17a presents the statistical distribution of the TC obtained from 500 Monte Carlo (MC) simulation runs. The circuit achieves a mean TC of 7.17 ppm/°C with a standard deviation of 4.13 ppm/°C. Figure 17b displays the results of 500 MC runs for the PSRR@1 Hz. Considering process variations and device mismatch, the mean PSRR is −128.6 dB with a standard deviation of 2.57 dB. Figure 17c illustrates the distribution of the output voltage *V_REF_*, showing a mean value of 3.0014 V and a standard deviation of 13.8 mV. Consequently, these comprehensive statistical analyses demonstrate the high precision and robust stability of the proposed voltage reference.

**Figure 18:**
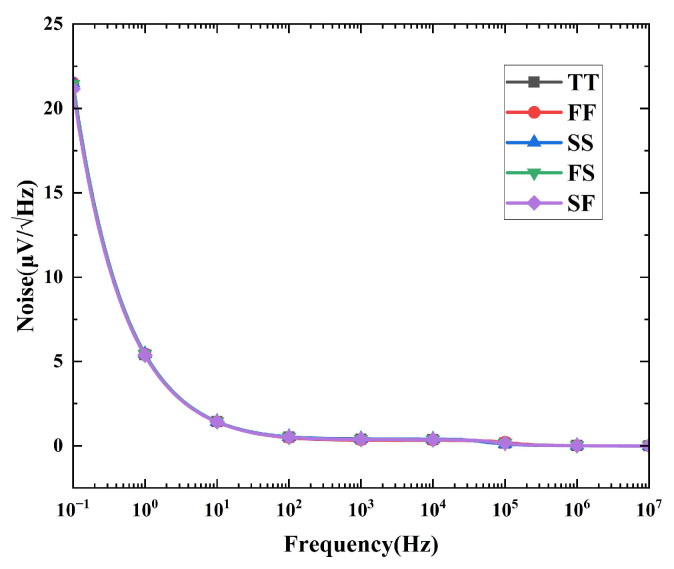
Output noise spectrum under different process corners.

Figure 18 illustrates the simulated output noise spectral density across five process corners. At 1 Hz, the noise density is 5.4 μV/√Hz under the TT corner.

**Figure 19:**
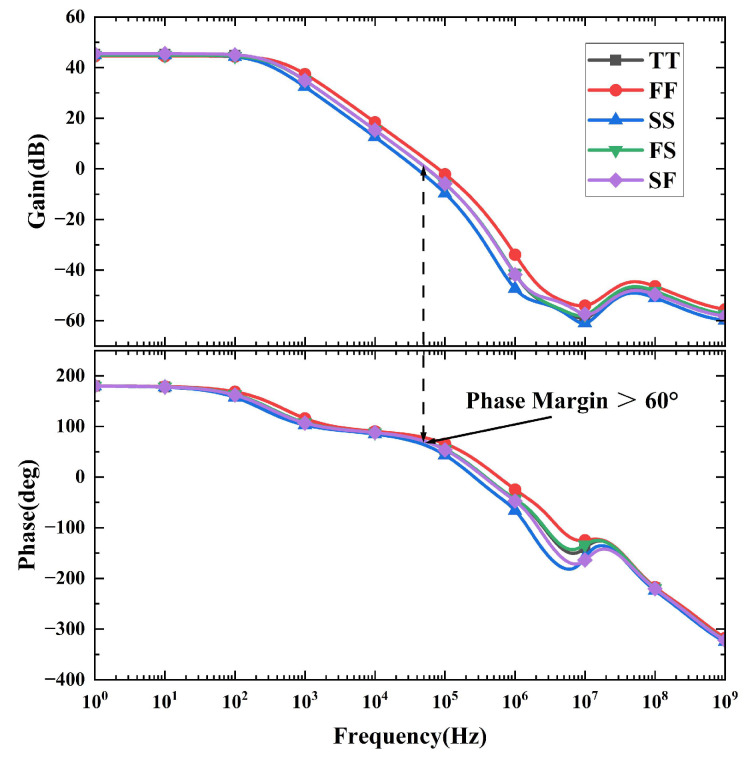
Loop gain and phase of the proposed voltage reference at different corners.

Figure 19 presents the simulated loop gain and phase response of the feedback loop across five process corners. The simulation results demonstrate that the phase margin of the bandgap reference circuit is maintained above 60° and ensures good loop stability.

Table 1 summarizes the performance of the proposed voltage reference and presents a comparison with recently published works. Compared with these reported designs, the proposed circuit achieves highly competitive performance in terms of three key metrics: temperature coefficient (TC), low-frequency PSRR, and line regulation.

## 4. Conclusions

This paper presents a high-precision voltage reference featuring low TC and high PSRR, designed for high-performance analog systems such as battery management systems (BMS). A base current correction technique is introduced to eliminate errors induced by the base current of BJTs. Simultaneously, a high-order compensation circuit is employed to effectively cancel the inherent nonlinearity of *V_BE_*, thereby achieving superior temperature stability. To suppress power supply noise, an improved FVF structure is utilized as a pre-regulator, providing a highly isolated internal supply for the core circuitry.

The proposed circuit is implemented in a standard 180-nm BiCMOS technology using 5 V devices to accommodate a 5.0 V supply voltage, occupying an active area of only 0.0459 mm^2^. Post-layout simulation results demonstrate that the circuit generates a reference voltage of 3.0 V. Over the temperature range of −40 °C to 125 °C, a minimum TC of 1.59 ppm/°C is achieved at the typical process corner. At room temperature (27 °C) with a 5.0 V supply, the quiescent current consumption is 23 μA. The design exhibits a PSRR of −128.89 dB at 1 Hz and maintains −102.9 dB up to 1 kHz. Furthermore, the line regulation is 0.00058 mV/V for a supply voltage range of 4.7 V to 5.3 V.

## Figures and Tables

**Table 1:** Performance comparison with recently published voltage references.

Parameter	[4]	[22]	[30]	[31]	[32]	This Work
Year	2024	2022	2018	2024	2025	2025
Technology (nm)	180	180	130	180	180	180
Supply voltage (V)	4.5–6	2.7–3.3	1.6	1.2–6	3.3	4.7–5.3
Supply current (μA)	4.2	<46	180	18.7	46	23
Reference voltage (V)	3.0	1.2	1.112	1.262	1.218	3.0
Temperature range (°C)	−40–120	−10–110	0–150	−50–130	−40–125	−40–125
TC (ppm/°C)	2.23	5–15	13.1	0.75	7.86	1.59
Line regulation (mV/V)	0.2	0.06	2.67	1.68	0.5846	0.00058
PSRR (dB)	−78.7@10 Hz	−80@DC	−40@10 Hz	−78@DC	−65@1 Hz	−128.89@1 Hz
Area (mm^2^)	0.104	0.448	0.1276	0.0079	0.01	0.0459
Simulated/Measured	Meas.	Meas.	Meas.	Sim.	Sim.	Sim.

## Data Availability

The original contributions presented in this study are included in the article. Further inquiries can be directed to the corresponding author.
